# Editorial: Technical and Methodological Advances in Proteomics

**DOI:** 10.3389/fchem.2021.795426

**Published:** 2021-12-20

**Authors:** Tara L. Pukala, Hao Chen

**Affiliations:** ^1^ Department of Chemistry, The University of Adelaide, Adelaide, SA, Australia; ^2^ Department of Chemistry and Environmental Science, New Jersey Institute of Technology, Newark, NJ, United States

**Keywords:** mass spectrometry, proteomics, chromatography, bioinformatics, ion mobility

Since the term proteomics was first coined in 1996 to describe the systematic study of the entire protein complement of a cell, tissue, or organism (Wilkins et al., 1996), advances in methods and technologies have propelled an explosion in the scope of biological studies. Consequently, proteomics has moved from reductionist biochemical analyses of isolated proteins to large-scale, parallelized characterization of the many and diverse properties of proteins, including expression, structures, interactions, localization and modifications at any given biological state. Driven by the realisation that proteins as the products of gene expression are fundamentally more complex and more directly associated with function than the gene itself, the goal of proteomics remains to obtain an integrated view of biology by studying the dynamic properties of all the proteins of a system rather than each individually.

In recent decades there has been a constant flow of technological advances that have enabled researchers to capture a deeper view of the proteome. With the development of 2D electrophoresis came the ability to map the complexity of a whole proteome (O’Farrell, 1975), however it wasn’t until sensitive protein-sequencing technologies became feasible that possibilities of proteomics would be fully realized. The sensitivity of analysis, analytical throughput and accuracy of protein identification by mass spectrometry (MS) has increased by orders of magnitude since initial applications to biomolecules. With continued advancements and coupling with complementary gas phase technologies such as ion mobility spectrometry, MS has become the mainstay analytical methodology in proteomic studies.

Advances in sample preparation have gone hand in hand with advances in instrumentation, with developments in protein extraction and derivatization methods and separation approaches such as electrophoresis and chromatography underpinning our ability to resolve complex protein mixtures into their individual components. This is particularly critical given the extreme diversity in structures afforded by post-translational modifications (PTMs) which have critical importance in modulating biological activity. At the other end of the experiment, obtaining biological insights requires interpretation of the often vast bodies of proteomics data. Hence improved methods of bioinformatics analysis are required to both better inform proteomic study design and evaluate and interpret the long list of protein identifications and abundances obtained from experiment to gain insight into biological function and regulation.

In this research topic, we have collated a selection of original research and review articles detailing new developments in technology, critical to the advancement of protein identification and structural characterization. These articles also demonstrate the increasing importance of separation and data analysis pipelines in optimising the information content afforded by proteomics experiments.

To emphasize the impact of sample preparation and chemical labelling methods in proteomics analyses, Kobayashi and Imai provide an overview of recent developments in fluorogenic derivatisation (FD) coupled with liquid chromatography (LC) for detection and quantification of proteins. They highlight its simplicity and reproducibility for protein separation and quantification prior to identification by traditional bottom-up MS approaches using a database search algorithm and demonstrate applications for comprehensive differential proteomics analysis. Finally, new developments for nano-LC-FD-LC systems and direct coupling to MS are described.

The importance of fractionation and separation methods has been further highlighted by Young et al., here in the context of investigating protein post-translational modifications. The significance of glycosylation in controlling protein function and dysfunction is widely acknowledged, as are the analytical challenges associated with MS structure determination of glycans, in part due to their isomeric structures and notable heterogeneity. This study establishes the utility of in-house packed porous graphitic carbon columns for the robust and reproducible LC-MS analysis of N-glycans, highlighting quantitative applications in biomarker discovery from ovarian cancer tissues.

Pang et al. build on the analysis of protein glycosylation in their study of monoclonal antibody biologics, where the glycosylation profile is key to optimal therapeutic outcomes and hence is a vital consideration in bio-manufacture quality control. They describe a robust, semi-automated workflow for glycoproteomic characterisation and quantitation from an LC-MS dataset of glycopeptides and peptides that is suitable to rapidly relay information back to the manufacturing line in biologic development scenarios, with limited user expertise and intervention. This work nicely highlights the importance of continued innovation in data analysis strategies to support integration of proteomics methodology into settings outside of the research environment.

Moving from untargeted analyses to targeted proteomics strategies can be a powerful approach for sensitive detection and precise quantification of selected proteins underpinning key biological processes. However, this is complicated in samples from complex, multi-species systems, where biochemical processes of interest are often controlled by many homologous proteins with varied amino acid sequences which confounds LC-MS identification and quantification. Medvecky and Mandalakis report a bioinformatic pipeline that overcomes this complexity for microbial systems by gathering homologous protein sets and extracting the most representative, process-specific and LC-MS/MS-amenable peptides for targeted analysis, to gain improved insights into microbial function.

Finally, Butler et al. demonstrate the power of proteomics approaches in the realm of structural biology. Ion mobility coupled with native MS can reveal insights into the tertiary and quaternary structures of proteins that may be intractable using traditional structural biology methods, particularly for heterogeneous systems. With a focus on Cu/Zn superoxide dismutase important in protection of cells from oxidative damage, they describe structural transitions along the unfolding pathway from the holo-dimer to the holo-monomer, single-metal monomer, and apo-monomer across multiple commercially available instrument platforms, and underscore the ability of this technology to provide valuable structural information for molecular modelling of protein structure and interactions.
